# Impact of Eprazole Sodium combined with somatostatin on treatment efficacy and coagulation function of patients with upper gastrointestinal bleeding: A retrospective study

**DOI:** 10.12669/pjms.41.10.12710

**Published:** 2025-10

**Authors:** Jingyang Du, Junpeng Xu, Sheng Xu, Murong Chen

**Affiliations:** 1 Jingyang Du Department of Emergency, The First People’s Hospital of Yongkang, Yongkang, Zhejiang Province 321300, P.R. China; 2 Junpeng Xu Department of Emergency, The First People’s Hospital of Yongkang, Yongkang, Zhejiang Province 321300, P.R. China; 3 Sheng Xu Department of Gastroenterology, The First People’s Hospital of Yongkang, Yongkang, Zhejiang Province 321300, P.R. China; 4 Murong Chen Dept. of Ultrasound,The Second People’s Hospital of Yongkang, Room 205, Building 3, Xingyue Garden, Yongkang, Zhejiang Province 321300, P.R. China

**Keywords:** Coagulation, Eprazole sodium, Omeprazole, Somatostatin, Upper gastrointestinal bleeding

## Abstract

**Objective:**

To investigate the impact of eprazole sodium combined with somatostatin on the coagulation function and treatment efficacy of patients with upper gastrointestinal bleeding.

**Methodology:**

In this single-center retrospective observational study, clinical data of 105 patients with upper gastrointestinal bleeding admitted to The First People’s Hospital of Yongkang (Jinhua, Zhejiang, China) from April 2023 to March 2025 were analyzed. Fifty-one patients who were treated with somatostatin and omeprazole comprised the control group. Fifty-four patients who received eprazole sodium in combination with somatostatin were included in the study group. The treatment effect, rehabilitation index (time of hemostasis, blood transfusion volume, length of hospitalization), coagulation function [fibrinogen (FIB), prothrombin time (PT), thrombin time (TT), activated partial thromboplastin time (APTT)] level, and adverse reaction rate were compared.

**Results:**

The total efficacy of the study group (96.30%) was higher than that of the control group (84.31%), while the duration of hemostasis, volume of blood transfusion, and length of hospital stay were lower (P<0.05). After treatment, the levels of FIB were higher, while PT, TT, and APTT levels decreased in both groups. The posttreatment levels of FIB in the study group were considerably higher than in the control group, while the levels of PT, TT, and APTT were significantly lower (*P*<0.05). The incidence of adverse reactions was comparable in the two groups (P>0.05).

**Conclusions:**

In patients with upper gastrointestinal bleeding, the combination of eprazole sodium and somatostatin can effectively shorten the hemostasis time, reduce the blood transfusion volume, improve the coagulation function, improve the overall treatment effect, promote early recovery and discharge without increasing the risk of adverse reactions.

## INTRODUCTION

If not treated, upper gastrointestinal bleeding, characterized by an acute onset and rapid progression, may cause hemorrhagic shock that endangers the life of the patient.^1^,^2^ With the gradual aging of the population, the increased incidence of cardiovascular diseases, and the increase in the use of non-steroidal anti-inflammatory drugs (NSAIDs), the incidence of upper gastrointestinal bleeding is on the rise.^3^,^4^ Therefore, developing strategies to quickly and effectively control bleeding and improve coagulation function in patients is crucial.

Proton Pump Inhibitors, such as omeprazole and inhibitory hormone, somatostatin, are routinely used in treating upper gastrointestinal bleeding.^5^ Omeprazole, as the first-generation proton pump inhibitor, can inhibit gastric acid secretion by specifically acting on gastric mucosal parietal cells, creating a suitable pH environment for coagulation and platelet aggregation, and promoting hemostasis.^6^,^7^ Somatostatin can effectively reduce visceral blood flow, decrease portal pressure, inhibit gastric acid and pepsin secretion, and thereby reduce stimulation to the bleeding site, achieving hemostasis.^8^,^9^ However, the potential impact of the combined treatment regimen on the homeostasis is still unclear.^10^

As a new generation of proton pump inhibitors, eprazole sodium has demonstrated to have a strong acid-inhibiting effect and long-lasting action, which can more effectively maintain the high pH value in the stomach and be a better choice for the treatment of upper gastrointestinal bleeding.^11^,^12^ At present, there is no large-sample, high-quality research on the clinical effect of eprazole sodium or omeprazole combined with somatostatin on the coagulation function of patients with upper gastrointestinal bleeding. Comparing the efficacy of different combined treatment schemes is of great practical significance for optimizing the treatment strategy of upper gastrointestinal bleeding, enhancing clinical treatment outcomes, and reducing patient mortality rates. The purpose of this study was to systematically compare the effect of eprazole sodium or omeprazole combined with somatostatin in the treatment of upper gastrointestinal bleeding through clinical observation. Evaluating the impact of combined therapy on patients’ coagulation function may provide a reliable reference for clinical treatment. Consistent with this objective, our analysis focused on overall clinical effectiveness of the regimens in upper gastrointestinal bleeding, rather than etiology-specific effects, given the limited subgroup sizes available in routine practice.

## METHODOLOGY

This was a single-center retrospective observational study conducted at The First People’s Hospital of Yongkang (Jinhua, Zhejiang, China) from April 2023 to March 2025. Clinical data of 105 hospitalized patients with upper gastrointestinal bleeding were included. Fifty-one patients received somatostatin plus omeprazole (control group), and fifty-four received somatostatin plus eprazole sodium (study group).

### Ethical approval:

Ethics Committee of the First People’s Hospital of Yongkang approved the study with the number 2025-LW-011, Date: May 19^th^ 2025.

### Inclusion criteria:

- Meet the diagnostic criteria^13^ for upper gastrointestinal bleeding.
- Trans gastroscopy, gastrointestinal barium meal and CT angiography.
- The age is 18~80.
- Systolic blood pressure ≧90mmHg, heart rate ≦ 120 times/min, relatively stable.
- Time from onset to admission <72 h.
- The patient or their legal guardian can understand the study content and sign a written informed consent form.
- Complete clinical data.

### Exclusion criteria:

- Patients with severe dysfunction of essential organs such as heart, liver and kidney.
- Patients with hematological diseases, such as thrombocytopenic purpura and hemophilia.
- Late-stage malignant tumor.
- Pregnant or lactating women.
- Persons with mental disorders who cannot correctly express their feelings.
- Patients who have undergone upper digestive tract surgery or other major surgery within one month.
- Patients who have a history of drug abuse or have used other drugs that affect coagulation or gastric acid secretion within the last one week.

### Somatostatin and omeprazole regimen (Control Group):

Patients received 250 micrograms of intravenous somatostatin (Zhejiang Asia Pacific Pharmaceutical Co., Shaoxing, China) as a loading dose, followed by continuous intravenous infusion at a rate of 250 μg/h. Additionally, 40 mg of omeprazole was administered intravenously twice daily, along with 100 ml of saline.

### Eprazole sodium in combination with somatostatin regimen (Study Group):

The dosage, mode, and course of somatostatin treatment were the same as those of the control group. Eprazole sodium (Lizhu Pharmaceutical, Zhuhai, Guangdong, China), 10 mg in 100 ml of saline, was administered intravenously once/daily. Treatment was continued for 3–5 days and the exact duration was individualized by the treating physician based on symptom control, hemodynamic stability, and laboratory reassessment. Most patients completed 4–5 days of therapy, whereas a minority with rapid symptom resolution discontinued after three days.

### Observational indices:

- The treatment effect of the two groups was calculated. Cured: vital signs returned to normal within 36 hours after treatment, fecal occult blood test results are negative, and the black stool and hematemesis disappear, indicating that the bleeding has stopped. Effective: vital signs return to normal 36~72 hour after the treatment, fecal occult blood test results are negative, hematemesis disappears, but there is still a small amount of black stool. Failed: After 72 hours of treatment, the vital signs did not return to normal, the fecal occult blood test results are positive, and the black stool and hematemesis did not improve; Cured and Effective were incorporated into the calculation of the Total efficacy.
- Two groups of rehabilitation indicators were calculated, including hemostasis time, blood transfusion volume, and length of hospital stay.
- The coagulation function of the two groups was assessed before and after treatment using 5- ml of fasting venous blood. The levels of fibrinogen (FIB), prothrombin time (PT), thrombin time (TT), and activated partial thromboplastin time (APTT) were detected by a full-automatic hemagglutinator.
- The incidence of adverse reactions, such as rash, vomiting and nausea, skin pruritus, gastrointestinal discomfort, dizziness, headache, etc., was compared.

### Safety assessment:

Adverse events (AEs) were monitored from admission to discharge by attending physicians during daily ward rounds and confirmed by chart review (progress notes, nursing records, medication administration records, and laboratory alerts). An AE was defined as any unfavorable and unintended sign, symptom, or disease temporally associated with treatment initiation, irrespective of causality. In this retrospective dataset, formal severity grading (e.g., CTCAE) was not performed. According to the medical records, no severe or life-threatening AEs occurred; all reported AEs were mild and self-limited, resolving spontaneously or with symptomatic management, and no treatment discontinuations were required.

### Missing data handling:

All 105 patients had complete data for the primary outcomes (therapeutic efficacy, coagulation parameters, and adverse events); therefore, analyses were conducted on a complete-case basis without imputation. A small number of secondary laboratory values (e.g., repeated hemoglobin at fixed time points) were not consistently available and were not included in the primary analyses.

### Statistical analysis:

SPSS 25.0 was used for all analyses. Measurement data were expressed as mean(*χ̅*±*S*), and the *t* test was used; Frequency and composition ratio (%) described counting data, and the Chi-squared test was used; P<0.05 indicated a statistically significant difference.

## RESULTS

There was no significant difference in the baseline data of the two groups (*P*>0.05) Table-I. The total efficacy of the study group (96.30%) was higher than that of the control group (84.31%) (*P*<0.05) (Table-II). Time to hemostasis, transfusion volume, and the total length of stay (LOS) in the study group were lower than in the control group(*P*<0.05) (Table-III).

**Table-I:** Baseline data.

Item	Study Group (n=54)	Control Group (n=51)	t/χ^2^ Value	P Value
***Sex [n(%)]***				
Male	32(59.26)	28(54.90)	0.203	0.652
Female	22(40.74)	23(45.10)		
Age(*X̄*±S, year)	58.51±9.02	56.96±8.78	0.891	0.375
Bleeding volume(*X̄*±S, ml)	602.15±54.71	599.91±60.25	0.200	0.842
Cause of bleeding[n(%)]				
Peptic ulcer	28(51.85)	25(49.02)	0.219	0.896
Esophageal and fundic varices rupture	21(38.89)	22(43.14)		
Others	5(9.26)	4(7.84)		
Course of disease(*X̄*±S, h)	1.89±0.41	1.92±0.37	0.393	0.695
***Alcohol use[n(%)]***				
+	15(27.78)	19(37.25)	1.076	0.300
-	39(72.22)	32(62.75)		
***Tobacco use[n(%)]***				
+	20(37.04)	16(31.37)	0.374	0.541
-	34(62.96)	35(68.63)		

**Table-II:** Comparison of treatment effect between two groups[n(%)].

Group	Number of cases	Markedly Effective	Effective	Ineffective	Total efficacy
Study Group	54	29(53.70)	23(42.59)	2(3.70)	52(96.30)
Control Group	51	22(43.14)	21(41.18)	8(15.69)	43(84.31)
χ^2^ Value					4.371
P Value					0.037

**Table-III:** Comparison of rehabilitation indices between two groups(*χ̅*±*S*).

Group	Number of cases	Hemostasis time(h)	Transfusion volume (ml)	LOS(d)
Study Group	54	9.30±2.69	160.52±10.29	8.18±1.35
Control Group	51	15.32±5.22	199.43±13.36	10.22±2.41
t Value		7.488	16.774	5.390
P Value		0.000	0.000	0.000

LOS: length of stay.

In terms of coagulation function (Table-IV), both groups demonstrated comparable pretreatment levels of FIB, PT, TT, APTT (P>0.05). After treatment, the levels of FIB were higher, while PT, TT, and APTT levels decreased in both groups. The posttreatment levels of FIB in the study group were considerably higher than in the control group, while the levels of PT, TT, and APTT were significantly lower (*P*<0.05). Notably, the mean PT (normal reference range: 9–13 s) and APTT (reference range: 23–35 s) values of both groups fell within the corresponding normal intervals after treatment, indicating that the improvements were not only statistically significant but also clinically relevant. The incidence of adverse reactions in the study group (3.70%) was comparable to that of the control group (7.84%) (*P*>0.05) (Table-V). No severe or life-threatening AEs were recorded; all reported AEs were mild and self-limited, and no treatment discontinuations occurred.

**Table-IV:** Comparison of Coagulation function between two groups(*χ̅*±*S*).

Period	Group	Number of cases	FIB(g/L)	PT(s)	TT(s)	APTT(s)
Before treatment	Study Group	54	2.39±0.64	15.78±2.25	21.77±3.37	35.50±4.37
	Control Group	51	2.43±0.56	16.07±2.14	22.08±3.06	34.86±5.09
	t Value		0.340	0.676	0.493	0.692
	P Value		0.735	0.501	0.623	0.490
After treatment	Study Group	54	3.91±0.70	10.77±1.36	7.79±1.57	24.36±3.74
	Control Group	51	3.54±0.62	11.94±1.56	9.55±2.76	26.77±4.21
	t Value		2.861	4.103	4.045	3.105
	P Value		0.005	0.000	0.000	0.003

FIB: fibrinogen; PT: prothrombin time; TT: thrombin time; APTT: activated partial thromboplastin time.

**Table-V:** Incidence of adverse reactions [n(%)].

Group	Number of cases	Rash	Vomiting nausea	Skin pruritus	Gastrointestinal discomfort	Dizziness headache	Overall incidence rate
Study Group	54	0(0.00)	1(1.85)	0(0.00)	1(1.85)	0(0.00)	2(3.70)
Control Group	51	1(1.96)	0(0.00)	2(3.92)	0(0.00)	1(1.96)	4(7.84)
χ^2^ Value							0.243
P Value							0.622

## DISCUSSION

Our findings indicate that eprazole sodium combined with somatostatin is safe and results in significantly higher overall efficacy and improved coagulation function compared with the omeprazole/somatostatin regimen.

The total efficacy of the study group in this report was 96.30%, which was significantly higher than that of the control group (84.31%) (P<0.05), indicating that eprazole sodium combined with somatostatin has a superior therapeutic effect on upper gastrointestinal bleeding. Somatostatin can reduce gastric acid and pepsin secretion, reduce visceral blood flow and portal pressure, and relieve the stimulation of the bleeding site.^14^ Omeprazole, as a first-generation proton pump inhibitor, inhibits gastric acid secretion and creates a suitable environment for coagulation;^15^ However, as a new generation of proton pump inhibitor, eprazole sodium has a more substantial and longer acid-inhibiting effect, and can better maintain the high pH value in the stomach.^16^

Synergistic action of somatostatin and eprazole can inhibit gastric acid secretion through multiple targets, reduce the erosion of gastric acid to the bleeding wound surface, accelerate the activation of prothrombin, thus significantly improving the treatment effect. The results of this study are consistent with the previous research. A study by Huang T et al.^17^, combined somatostatin with eprazole in patients with peptic ulcer and showed that the total efficacy of the combined treatment reached 93.33%, which was consistent with the high efficacy of the study group in our study. In contrast, Duan L et al.^18^ found that the total efficacy of acute upper gastrointestinal bleeding treated with a single proton pump inhibitor was only 83.67%, which was lower than the total efficacy of the study group in this study. This markedly increased efficacy of the combined regimen further confirms that the synergistic effect of both drugs can effectively enhance the treatment outcomes of upper gastrointestinal bleeding, possibly through their stronger acid-inhibiting ability and longer-lasting action time. Combined eprazole sodium/somatostatin regimen, therefore, provides more effective options for clinical treatment and helps to improve the recovery rate of patients with upper gastrointestinal bleeding.

The eprazole sodium/somatostatin regimen was also associated with faster bleeding control, reduced blood loss, shorter hemostasis duration, lower transfusion requirements, and reduced hospital stay. Rapid hemostasis not only reduces the risk of complications caused by blood loss and promotes the rehabilitation of patients, but also shortens the hospital stay, reduces the economic burden, and the risk of hospital infection. A study of Ming et al.^19^ found that somatostatin combined with omeprazole could shorten the time of hemostasis. This study demonstrated that the time to hemostasis was shorter for patients who received a combination of eprazole and somatostatin, possibly due to the more stable high intragastric pH environment maintained by eprazole sodium, which not only promotes coagulation but also reduces the risk of rebleeding and accelerates patient recovery.

After the treatment, FIB levels increased and PTs, TTs, and APTTs decreased in both groups, and improvements were more pronounced in the study group (*P*<0.05). Importantly, the mean PT and APTT values in both groups normalized to within standard laboratory reference ranges (PT: 9–13 s; APTT: 23–35 s). This indicates that the observed improvements were not only statistically significant but also clinically meaningful, reflecting restoration of coagulation function toward normal physiology. These results suggest that sodium eprazole in combination with somatostatin is more effective in improving coagulation in patients. Elevated levels of fibrin are beneficial for the formation of stable fibrin clots.^20^ The shortening of PTs, TTs, and APTTs suggests that combination therapy accelerates the coagulation process. Eprazole sodium maintains a high intragastric pH environment, promotes the activation of coagulation factors, and reduces inhibition of coagulation by gastric acid.^21^ The combined eprazole sodium/somatostatin treatment significantly improved FIB, PT, TT, and APTT levels, underscoring its superior efficacy in regulating coagulation and reducing the risk of rebleeding in patients with upper gastrointestinal bleeding. These results may provide new insights and approaches for the clinical management of upper gastrointestinal bleeding, ultimately improving patients’ prognosis.

The study did not detect a significant difference in the incidence of adverse reactions between the study group and the control group, indicating that the safety of eprazole sodium or omeprazole in combination with somatostatin was equally good. This finding is beneficial for enhancing patient compliance and ensuring the smooth progression of treatment.

The observed difference in dosing frequency between regimens is pharmacologically driven rather than a design-related confounder. Eprazole sodium exhibits more sustained inhibition of gastric acid secretion, enabling once-daily administration to maintain a therapeutic intragastric pH conducive to clot stability. By contrast, omeprazole has a shorter effective duration and is commonly administered twice daily to achieve comparable acid suppression. Hence, part of the clinical advantage observed with eprazole sodium may be attributable to its prolonged pharmacodynamic effect and a simplified dosing scheme, which can streamline inpatient workflows (e.g., fewer infusions) without deviating from standard care.

Current international and national guidelines for upper gastrointestinal bleeding recommend early use of proton-pump inhibitors for non-variceal bleeding and vasoactive agents for suspected or confirmed variceal bleeding, together with timely endoscopy.^22^ The present regimen—eprazole sodium plus somatostatin—fits within this framework: the PPI component sustains an intragastric pH favorable for hemostasis, whereas somatostatin reduces splanchnic blood flow and portal pressure. Our findings therefore reflect guideline-concordant pharmacotherapy rather than a departure from standard care. Notably, drug therapy is complementary to, and not a substitute for, endoscopic hemostasis where indicated. Although a formal pharmacoeconomic evaluation was not prespecified, once-daily dosing of eprazole sodium may reduce nursing time and infusion-pump occupancy compared with twice-daily PPI regimens. The observed reductions in time to hemostasis and length of stay in our cohort suggest potential downstream cost savings; however, these hypotheses require confirmation using cost-effectiveness models and prospective pragmatic studies. From an implementation standpoint, the regimen is readily deployable in tertiary centers and remains feasible in secondary or primary settings where endoscopic capacity may be limited or delayed. The dosing scheme is simple, monitoring relies on routine parameters (vital signs, hemoglobin, coagulation), and no additional devices are required beyond standard intravenous access. In resource-constrained sites, the regimen may serve as initial stabilization while arranging definitive endoscopy.

### Limitations:

Although this study demonstrates the efficacy and safety of eprazole sodium combined with somatostatin in the treatment of upper gastrointestinal bleeding, several limitations should be noted. First, as a retrospective single-center study, selection bias and residual confounding cannot be excluded, and the relatively small sample size may limit the generalizability of the findings. Second, adverse event (AE) severity was not graded using standardized criteria (e.g., CTCAE); although no severe AEs occurred and all events resolved with symptomatic care, future prospective studies should adopt formal grading frameworks to improve the rigor and comparability of safety assessment. Third, treatment efficacy was mainly evaluated by clinical signs (e.g., melena resolution and vital sign stabilization) rather than validated scoring systems such as Rockall or Glasgow-Blatchford, which were not consistently recorded in this dataset. Fourth, only short-term outcomes were assessed, while long-term efficacy and recurrence remain to be confirmed. Fifth, we did not perform etiology-specific subgroup analyses or multivariable modeling due to limited events, which could have led to unstable estimates; larger multi-center prospective studies with stratified and adjusted analyses are warranted. Finally, no cost-effectiveness evaluation was conducted, and variations in drug acquisition costs and resource utilization across hospitals may affect the generalizability of economic implications.

## CONCLUSION

The combination of eprazole sodium and somatostatin is safe and can effectively shorten the time of hemostasis, reduce the amount of blood transfusion, improve coagulation function, enhance the overall treatment effect, promote patient early recovery and discharge, without increasing the risk of adverse reactions.

### Authors’ contributions:

**JD:** Literature search, study design and manuscript writing.

**JX, SX and MC:** Data collection, data analysis and interpretation. Critical Review.

**JD:** Manuscript revision and validation and is responsible for the integrity of the study.

All authors have read and approved the final manuscript.
